# Transcripts Expressed during Germination *Sensu Stricto* Are Associated with Vigor in Soybean Seeds

**DOI:** 10.3390/plants11101310

**Published:** 2022-05-14

**Authors:** Karina Renostro Ducatti, Thiago Barbosa Batista, Welinton Yoshio Hirai, Daiani Ajala Luccas, Leticia de Aguila Moreno, Cristiane Carvalho Guimarães, George W. Bassel, Edvaldo Aparecido Amaral da Silva

**Affiliations:** 1 Department of Crop Science, College of Agricultural Sciences, São Paulo State University (UNESP), Botucatu 18610-034, SP, Brazil; karinaducatti@hotmail.com (K.R.D.); batista.thiagob@gmail.com (T.B.B.); ajaladaiani@gmail.com (D.A.L.); ldamoreno@uga.edu (L.d.A.M.); criscgbiologia@hotmail.com (C.C.G.); 2 Department of Exacts Sciences, College of Agriculture “Luiz de Queiroz”, University of São Paulo, Piracicaba 13416-000, SP, Brazil; wyhirai@gmail.com; 3 School of Life Sciences, The University of Warwick, Coventry CV4 7AL, UK; george.bassel@warwick.ac.uk

**Keywords:** germinative process, seedling establishment, molecular tools

## Abstract

The rapid and uniform establishment of crop plants in the field underpins food security through uniform mechanical crop harvesting. In order to achieve this, seeds with greater vigor should be used. Vigor is a component of physiological quality related to seed resilience. Despite this importance, there is little knowledge of the association between events at the molecular level and seed vigor. In this study, we investigated the relationship between gene expression during germination and seed vigor in soybean. The expression level of twenty genes related to growth at the beginning of the germination process was correlated with vigor. In this paper, vigor was evaluated by different tests. Then we reported the identification of the genes *Expansin-like A1*, *Xyloglucan endotransglucosylase/hydrolase 22*, *65-kDa microtubule-associated protein*, *Xyloglucan endotransglucosylase/hydrolase 2*, *N-glycosylase/DNA lyase OGG1* and *Cellulose synthase A catalytic subunit 2*, which are expressed during germination, that correlated with several vigor tests commonly used in routine analysis of soybean seed quality. The identification of these transcripts provides tools to study vigor in soybean seeds at the molecular level.

## 1. Introduction

Soybean (*Glycine max* (L.) Merrill.) (2*n* = 40 chromosomes) is a herbaceous and autogamous plant belonging to the Fabaceae family. Soybean production accounts for 56% of the world oilseed production [1]. It is considered a global commodity and is highly affected by biotic and abiotic factors [2]. Thus, obtaining soybean seeds with high physiological quality which germinate uniformly and rapidly is paramount for the success of the production of this oilseed. Seeds with superior physiological quality are the input for agriculture [3] because they are responsible for the successful establishment of the desired stand, which is essential to achieve high productivity [4].

Vigor is an important component of the physiological quality [5] and is responsible for the seed’s resilience [6]. Thus, vigorous seeds present better physiological performance under non-ideal conditions for their germination (capacity for the new individual to grow from the embryo), and/or germinate faster according to Fonseca de Oliveira et al. [3] and Ebone et al. [4], which leaves the seed exposed to adverse conditions for a shorter time. The most robust and accepted definition of vigor is ‘the sum total of those properties of the seed that determine the potential level of activity and performance of the seed during germination and seedling emergence’ [7,8]. In this sense, vigorous seeds have a faster and more uniform seedling establishment [9], which contributes to the initial growth of the plants. It is important to highlight that, seeds with similar germination may have variation in their vigor. This makes the germination analysis (mandatory for its commercialization) insufficient to determine the seed lots with the highest level of vigor. This is due to the germination test only evaluating the number of individuals with normal characteristics in an isolated way, at a certain period after sowing. Therefore, the screening of this property is commonly performed using measurements of germination speed, seedling growth and tolerance to stress, since these characteristics are more pronounced in seeds with a higher level of vigor. In parallel, to help in the diagnosis of more vigorous seeds, other tests can be used, such as membrane integrity, lipid peroxidation level and more recently the use of spectral images that map seed constituents [3,10].

In soybean, vigor is known as a physiological quality attribute that contributes to the increase in crop yield [4]. In this species, the acquisition of this property occurs progressively after the acquisition of desiccation tolerance at stage R7.1.3 in the maturation phase [11]. Despite the knowledge of its importance and of the time when vigor is acquired, research has been incipient in elucidating the molecular mechanisms underlying its expression in soybean seeds. Apparently, this is due to an overlap of physiological and molecular events in the temporal pattern of acquisition of physiological quality in soybean seeds during the maturation and late maturation phases [11,12]. This makes it difficult to separate and characterize the events that contribute to the acquisition of vigor at the molecular level.

Faced with the difficulty of studying vigor at the molecular level when it is acquired during maturation, an alternative is to study it during the germination process, i.e., when this property manifests itself and provides the establishment of seedlings quickly or under adverse conditions. This would be possible since the germination process has been well explored in relation to the association of its physiological and molecular mechanisms. For example, several groups of genes have been related to germination *sensu stricto* (the first stage of the germination process), mainly those involved with DNA repair, such as oxidative stress defense, cell respiration and hydrolysis of reserves, and cell elongation and expansion [13,14,15,16,17]. At the same time, the synchrony between the processes promotes a chain reaction that culminates in the growth of the embryonic axis, which leads to the occurrence of visible germination, i.e., growth of the new individual from the embryo, through the protrusion of the radicle due to the elongation of the transition zone between the hypocotyl and the radicle [18] as occurring in soybean embryos [19].

Recently, Ribeiro-Oliveira et al., [6] showed that the process that occurs during germination *sensu stricto* has a positive linear influence on vigor in soybean seeds. In other words, vigor is the key for the efficiency of the germination process, and the consequence of this is observed in the establishment of the seedling (quickly and under adverse conditions). Thus, theoretically, the study of the expression of genes related to growth acting during germination may reveal transcripts associated with seed vigor. One way to explore this would be using seeds with similar germination rates, but with differences in their vigor. Therefore, to test our hypothesis, we used seeds with differences in their vigor levels, previously screened from two commercial cultivars (cv1 and cv2) divided two lots (lot 1 and lot 2). Our aim was to verify if there was a correlation between the genes expressed during germination *sensu stricto* with seed vigor, evaluated by different properties related to this, i.e., speed germination, stress tolerance, organ elongation of seedlings, membrane integrity and tissue deterioration.

## 2. Results

### 2.1. Characterization of Seed Properties 

**Figure 1:**
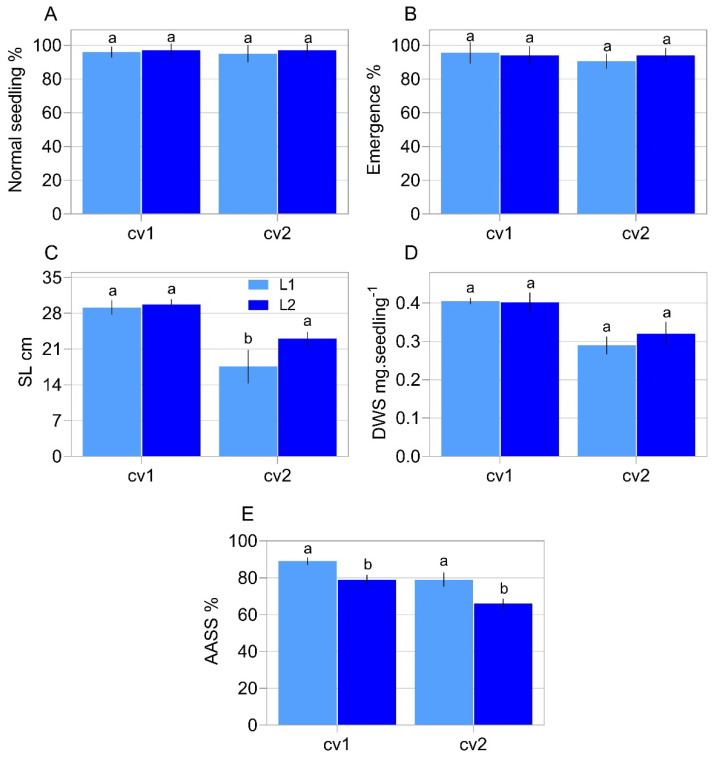
Physiological properties in two commercial cultivars (cv1-BRS133 and cv2-MG/BR 46 (Conquista)) and their respective seed lots (L1: lot 1 and L2: lot 2). Normal seedlings (**A**), seedling emergence (**B**), seedling length (**C**), seedling dry weight (**D**) and accelerated ageing using saline solution–AASS (**E**). Different letters indicate a significant difference at the 5% probability level (*p* < 0.05). Error bars show standard deviation (*n* = 8).

Initially, we screened the physiological quality of our samples through several tests related with germination and vigor for commonly used soybean seeds. We noticed that normal seedlings and emergence did not differ between the lots of cultivars analyzed (Figure 1A,B). There was no difference regarding the length of seedlings of cv1 (Figure 1C). As for cv2, seedlings from lot 2 were longer in relation to those of lot 1 (Figure 1C). The seedling dry mass data were not influenced (Figure 1D). After accelerated aging there was a reduction in the ability to generate normal seedlings in seeds from lot 2 in all the cultivars analyzed (Figure 1E). After aging, the water content ranged from 13.2 to 13.5% between our seed samples, indicating that the moisture content did not influence the results obtained, since it did not change more than 0.5% between the samples.

**Figure 2:**
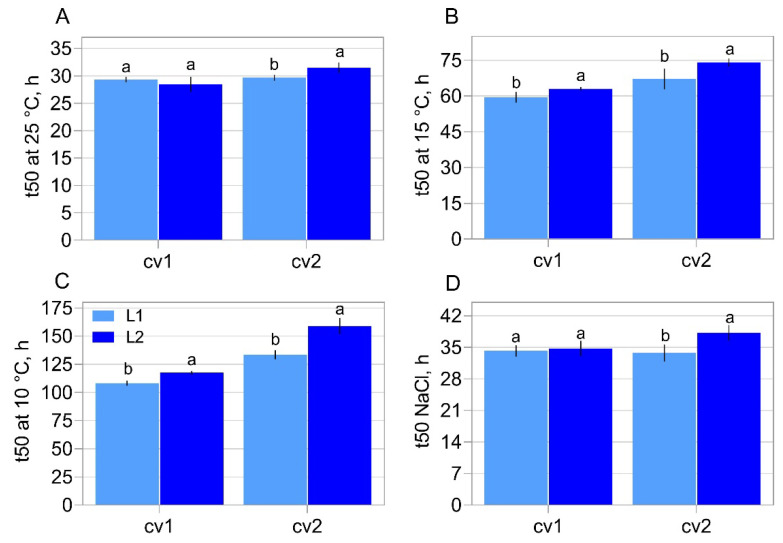
Time to reach 50% of root protrusion (t50) in two commercial cultivars (cv1-BRS133 and cv2-MG/BR 46 (Conquista)) and their respective seed lots (L1: lot 1 and L2: lot 2). t50 at 25 °C (**A**), 15 °C (**B**) and 10 °C (**C**), and t50 in saline substrate at 100 mmol.L^−1^ of NaCl (**D**). Different letters indicate a significant difference at the 5% probability level (*p* < 0.05). Error bars show standard deviation (*n* = 8).

Excluding the results of time to reach 50% of germination (t50) at 25 °C with and without the use of saline solution in the germination substrate for cv1, in all other conditions the seeds of lot 2 took longer to reach 50% of germination than the seeds of lot 1 (Figure 2). In general, as the test conditions became more adverse for germination, the differences between the lots became more evident, and at 10 °C and 15 °C, the t50 results showed significant differences between all the samples analyzed (Figure 2 B,C).

**Figure 3:**
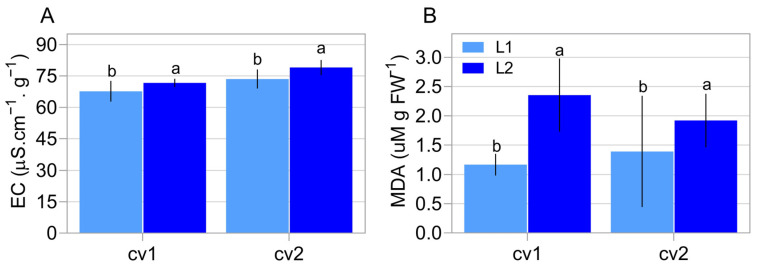
Biochemical properties in two commercial cultivars (cv1-BRS133 and cv2-MG/BR 46 (Conquista)) and their respective seed lots (L1: lot 1 and L2: lot 2). Electric conductivity (**A**) and Estimation of lipid peroxidation (MDA) (**B**). Distinct letters indicate a significant difference at the 5% probability level (*p* < 0.05). Error bars show standard deviation (*n* = 8).

Electrical conductivity (Figure 3A) showed higher amounts of leachate in the seeds of lot 2 of cv1 and cv2. Finally, malonaldehyde analysis showed that seeds from lot 2 had a higher estimated level of lipid peroxidation (Figure 3B).

**Figure 4:**
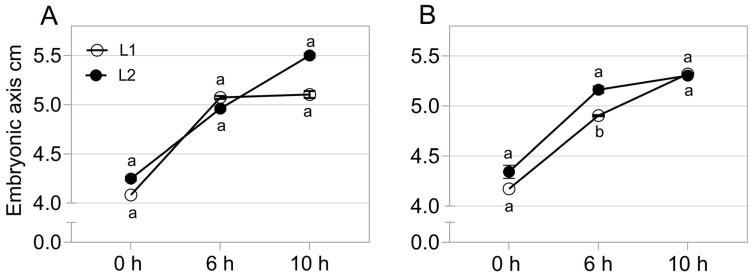
Embryonic axis length during germination in two commercial cultivars (A: cv1-BRS133 and B: cv2-MG/BR 46 (Conquista)) and their respective seed lots (L1: lot 1 and L2: lot 2) during germination *sensu stricto*. cv1 (**A**) and cv2 (**B**) at 0 h, 6 h and 10 h of germination. Distinct letters in each point of evaluation indicate a significant difference at the 5% probability level (*p* < 0.05). Error bars show standard deviation (*n* = 6).

There was an increase of 25% in the average length of the embryonic axis during germination *sensu stricto*, and the dynamics of growth did not change depending on the cultivar and its respective lots (Figure 4).

### 2.2. Gene Expression Related to Growth in the Embryonic Axis during Germination

**Figure 5:**
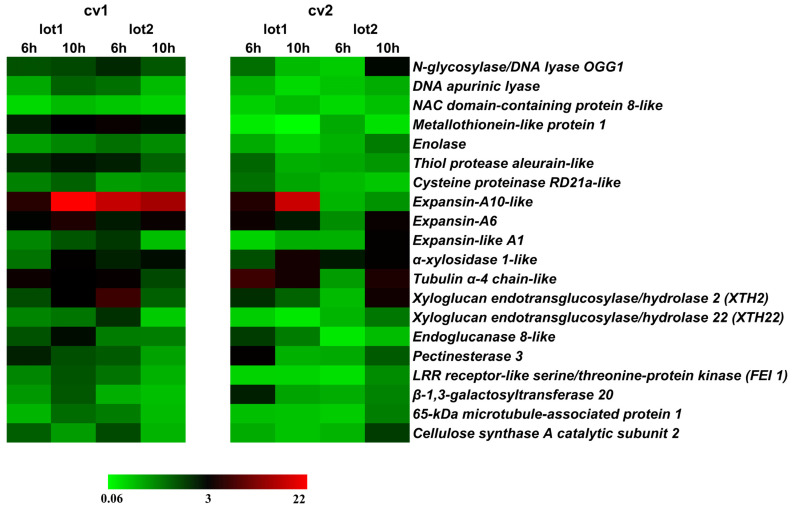
Heatmap of relative expression (2^^−ΔΔCT^) during germination *sensu stricto* in the embryonic axis in two commercial cultivars (cv1-BRS133 and cv2-MG/BR 46 (Conquista)) and their respective seed lots (L1: lot 1 and L2: lot 2). The relative expression was obtained using the Pair-Wise Fixed Reallocation Randomization Test (*n* = 6) in relation to the expression of dry embryonic axis.

In sequence, to further test our idea we explored gene expression during germination *sensu stricto* in previously characterized seeds with distinct physiological potential of each cultivar (Figure 5). Among the genes involved in DNA repair, the relative expression of the gene encoding *N-glycosylase/DNA lyase OGG1* stands out, which was twice as high at 6 h and 10 h in the germination of seeds from lots 1 and 2 of cv1. The seeds of lot 2 from cv2 showed a decrease in expression after 6 h of imbibition, with a greater expression at 10 h. In seeds from cv1 the expression of the *DNA apurinic lyase* expression was more accentuated at 10 h in the seeds of L1 and at 6 h in the seeds of L2 (Figure 5).

The relative expression of *Metallothionein-like protein 1* (*MT1*) was high along germination for seed lots 1 and 2 of cv1. The seeds of lot 1 from cv2 showed a decrease in three times on *MT1* expression in the beginning of germination (Figure 5). The expression of *enolase*, an enzyme involved in the glycolytic pathway, was observed in the embryonic axis of soybean seeds and was not significant for any of the cultivars and their respective lots (Figure 5).

Regarding the reserve mobilization process, it was observed in seeds from lot 1 of cv1 that the relative expression of *Thiol protease* in the embryonic axis increased by 3 × and that *Cysteine proteinase* increased by 2 × 10 h after beginning germination. For seeds from lot 2 of cv1, the level of *Thiol protease* was highest at 6 h. In cv2 seeds, there was no variation in the relative expression of these transcripts during germination *sensu stricto* and among seeds of the studied lots (Figure 5).

The relative expression of *expansin-A10-like* (*EXPA10*), *expansin-A6* (*EXPA6*) and expansin-like A1 (*EXPA1*) progressively increased in seeds from lot 1 of cv1. In the seeds of lot 2, however, there was a similar pattern of the increase profile only for *EXPA10*, which was 18 × greater than from seeds of lot 1. Reductions were observed along germination for *EXPA6* and *EXPA1*. In cv2 seeds, there was an increase for *EXPA10* and *EXPA6* in lot 1 seeds, while in lot 2 the tendency was for a reduction in the expression with advance of the germination process (Figure 5).

As observed for expansins, the relative expression of transcripts *α-xylosidase 1-like* (*XYL1*), *Tubulin α-4 chain-like* (*TUA*), *Xyloglucan endotransglucosylase/hydrolase 2* (*XTH2*), *Endoglucanase 8-like* (*EGase*), *Pectinesterase 3* (*PME*) and *LRR receptor-like serine/threonine-protein kinase FEI* increased throughout germination *sensu stricto* in seed lot 1 of cv1. For cv2 seeds, the variations in the relative expression of transcripts were more accentuated during germination, in which, in general, lot 1 seeds showed greater expression at 6 h, while lot 2 seeds at 10 h after the beginning of germination (Figure 5), which suggest a delay in the expression of the genes mentioned earlier in these seeds.

The expression of *β-1,3-galactosyltransferase 20* increased strongly at 6 h of germination in lot 1 of cv2 and reduced afterwards. For cv2, although apparently showing the same trend, this increase was not observed in the same proportion of cv1 seeds (Figure 5).

In general, for *65-kDa microtubule-associated protein 1 gene*, an increase of two times in the expression during germination in our samples was verified. (Figure 5). An increase in the expression of *Cellulose synthase A catalytic subunit 2* occurred at 6 h in seeds from lot 1 and lo2 of cv1. For cv2 the expression was more accentuated only at 10 h in seeds of lot 2 (Figure 5).

Regarding the gene expression divergence between the cultivars used in this study, our expression analysis demonstrated that, in general, cv1 seeds have superior expression of the genes studied during the germination process, especially those belonging to lot 1 (Figure 5).

### 2.3. Association between Gene Expressions during Germination Sensu Stricto with Seed Vigor

Finally, considering the natural divergence in vigor of our samples and the changes in the expression profile of transcripts during germination as previously screened, we sought to find the genes most associated with the expression of seed vigor, here evaluated by different tests. For this, the cultivars and their respective lot parameters were analyzed jointly. Initially, we explored whether there were differences in the association of gene expression analyzed at 6 h and 10 h during germination *sensu stricto* with aspects of vigor. To this end, the bootstrap method was performed with 10,000 resamplings, using the gene expression of each sample obtained at 6 h and 10 h during germination in relation to the aspects of seed vigor (Figure 1, Figure 2 and Figure 3); and for these two bootstrap resamplings (6 h and 10 h) the analysis of regulatory canonical correlation (RCC) was applied. Thus, a mean correlation >0.7 was verified for the two process (Supplementary Figure S1), corroborating that the association of gene expression with seed vigor is equal for the different germination times analyzed. Thus, for subsequent analyses, the expression samples at 6 h and 10 h were used jointly to find associations with seed vigor.

**Figure 6:**
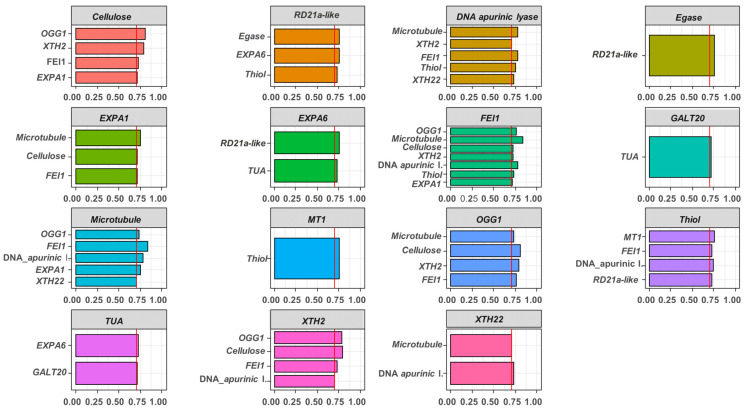
Correlation between genes analyzed during germination *sensu stricto* in seed embryos of two cultivars and their respective lots. Spearman method was used to obtain correlation coefficient (*r*) (*p* ≤ 0.05). Only values of *r* > 0.70 were presented. *N-glycosylase/DNA lyase OGG1* (*OGG1*), *DNA apurinic lyase*, *Metallothionein-like protein 1* (*MT1*), *Thiol protease aleurain-like* (*Thiol*), *Cysteine proteinase RD21a-like* (*RD21a-like*), *Tubulin alpha-4 chain-like* (*TUA*), *Expansin-like A1* (*EXPA1*), *Expansin-A10-like* (*EXPA10*), *Expansin-like A6* (*EXPA6*), *Xyloglucan endotransglucosylase/hydrolase 22* (*XTH22*), *Xyloglucan endotransglucosylase/hydrolase 2* (*XTH2*), *Endoglucanase 8-like* (*Egnase*), *LRR receptor-like serine/threonine-protein kinase FEI* (*FEI*1), *β-1,3-galactosyltransferase 20* (*GALT20*), *65-kDa microtubule-associated protein* (*microtubule*) and *Cellulose synthase A catalytic subunit 2* (*cellulose*).

Correlation coefficients indicated the existence of associations between the genes analyzed in the study, and notably certain genes analyzed were related to one or more distinct genes through high and positive correlation (*r* > 0.70). As an example, there is the *N-glycosylase/DNA lyase OGG1* transcript with a DNA repair function, which positively interacts with cell wall relaxation genes (*65-kDa microtubule-associated protein*, *Xyloglucan endotransglucosylase/hydrolase 2*, *Cellulose synthase A catalytic subunit 2* and *LRR receptor-like serine/threonine-protein kinase FEI*) (Figure 6).

**Figure 7:**
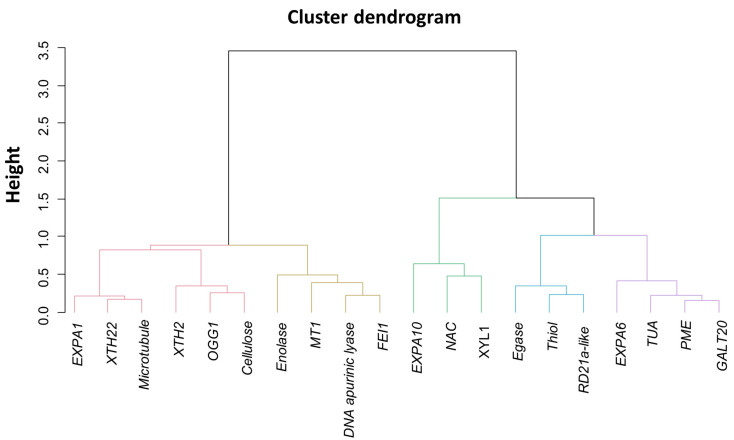
Gene clustering analyzed in soybean seed embryos during germination *sensu stricto* of two cultivars and their respective lots. *N-glycosylase/DNA lyase OGG1* (*OGG1*), *DNA apurinic lyase*, *NAC domain-containing protein 8-like* (*NAC*), *Metallothionein-like protein 1* (*MT1*), *Enolase*, *Thiol protease aleurain-like* (*Thiol*), *Cysteine proteinase RD21a-like* (*RD21a-like*), *Alpha-xylosidase 1-like* (*XYL1*), *Tubulin alpha-4 chain-like* (*TUA*), *Expansin-like A1* (*EXPA1*), *Expansin-A10-like* (*EXPA10*), *Expansin-like A6* (*EXPA6*), *Xyloglucan endotransglucosylase/hydrolase 22* (*XTH22*), *Xyloglucan endotransglucosylase/hydrolase 2* (*XTH2*), *Endoglucanase 8-like* (*Egnase*), *Pectinesterase 3* (*PME*), *LRR receptor-like serine/threonine-protein kinase FEI* (*FEI1*), *Beta-1,3-galactosyltransferase 20* (*GALT20*), *65-kDa microtubule-associated protein* (*microtubule*), *Cellulose synthase A catalytic subunit 2* (*cellulose*).

Faced with the undeniable correlation between the genes analyzed (Figure 6), we sought to group them according to an expression pattern. For this, a clustering of these was performed, applying a homogeneity criterion in the decomposition of the singular values of the first principal component (data not shown). Thus, the relationships between genes were maximized [20] enabling us to separate them into five different groups (Figure 7).

**Figure 8:**
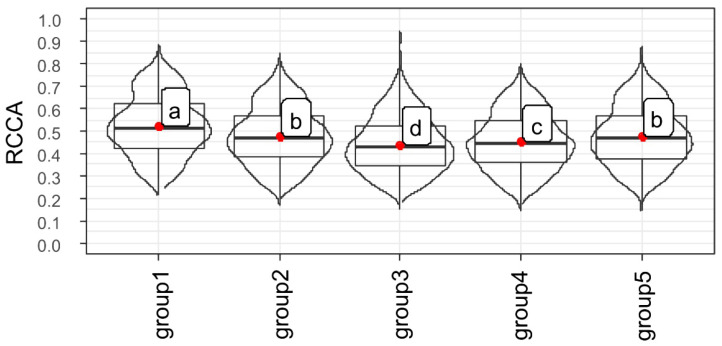
First eigenvalue of the Regulatory canonical correlation (RCCA) from the computational process Bootstrap 10,000 generated from each gene group (1, 2, 3, 4 and 5) with aspects of soybean seed vigor expression. Distinct letters indicate a significant difference at the 5% probability level (*p* < 0.05). Group 1: *N-glycosylase/DNA lyase OGG1* (*OGG1*), *Expansin-like A1* (*EXPA1*), *Xyloglucan endotransglucosylase/hydrolase 22* (*XTH22*), *Xyloglucan endotransglucosylase/hydrolase 2* (*XTH2*), *65-kDa microtubule-associated protein* (*microtubule*), *Cellulose synthase A catalytic subunit 2* (*cellulose*). Group 2: *DNA apurinic*, *Metallothionein-like protein 1* (*MT1*), *Enolase*, *LRR receptor-like serine/threonine-protein kinase FEI* (*FEI1*). Group 3: *NAC domain-containing protein 8-like* (*SOG1*), *Expansin-A10-like* (*EXPA10*), *Alpha-xylosidase 1-like* (*XYL1*). Group 4: *Endoglucanase 8-like* (*Egnase*), *Cysteine proteinase RD21a-like* (*RD21a-like*), *Thiol protease aleurain-like* (*Thiol*). Group 5: *Tubulin alpha-4 chain-like* (*TUA*), *Expansin-like A6* (*EXPA6*), *Pectinesterase 3* (*PME*), *Beta-1,3-galactosyltransferase 20* (*GALT20*).

The bootstrap re-sampling process was performed for each clustered group (Figure 7) with the aspects of vigor expression (Figure 1, Figure 2, Figure 3 and Figure 4), and the canonical correlation were estimated in each process generated by this re-sampling and expressed in Boxplots (Figure 8). Thus, it was possible to verify that the genes *Expansin-like A1*, *Xyloglucan endotransglucosylase/hydrolase 22*, *65-kDa microtubule-associated protein*, *Xyloglucan endotransglucosylase/hydrolase 2*, *N-glycosylase/DNA lyase OGG1* and *Cellulose synthase A catalytic subunit 2* (group 1) have a greater correlation with the expression of seed vigor (Figure 8). This demonstrated that of the twenty genes studied, only six were strongly associated with the vigor properties of soybean seeds (Figure 7 and Figure 8).

## 3. Discussion

Here we studied the expression of genes associated with growth during germination *sensu stricto* and traced their association with the expression of the seed vigor evaluated. We wanted to take a step towards understanding the physiological expression of vigor in soybean seeds and, in parallel, propose the use of certain transcripts, among those studied here, as a tool to assess seed vigor at a molecular level.

It was noted that the germination and emergence rate had not changed depending on the lot (Figure 1A,B). However, the vigor tests demonstrated a clear distinction in the vigor level between the lots of each cultivar, regularly marked by the accelerated aging test and the germination speed (t50) (Figure 1E and Figure 2). The difference in the vigor levels of the analyzed lots was confirmed by the divergence regarding their lipid peroxidation (Figure 3B), which can be considered an indication of the “health status” of the seeds, where a higher concentration of peroxides indicates more advanced tissue deterioration. In addition, the seeds of cv1 demonstrated superior vigor compared to the seeds of cv2 (Figure 1E, Figure 2 and Figure 3). Thus, the seeds used in this study have different ways of performing the germination process. This characterizes divergence in their vigor, our target phenomena.

An increase of 25% of the embryonic axis length during germination was observed (Figure 4). This occurred as a result of events involved in germination *sensu stricto*. Among these, is the beginning of cell elongation, responsible for boosting radicle protrusion. This process depends on the expression of genes involved in cell wall modification and relaxation, such as expansins (*EXP*), *xyloglucan endotransglycosylase/hydrolases* (*XTH*), *pectin methyl esterases* (*PME*) and *β-1,4-glucanases* [13], which jointly with genes associated to DNA repair, were targeted in our study. The expression of these transcripts changed throughout the germinative process (Figure 5) due to the signaling necessary for the growth of the embryonic axis. According Smolikova et al. [21], this phase is marked by rearrangements of signaling pathways and a switching of gene expression programs. This explains the intercorrelations between the genes here evaluated, by demonstrating the interactivity between processes necessary for the occurrence of germination (Figure 6), i.e., different signals are required for its success.

Among the target genes analyzed in our study, *Expansin-like A1*, *Xyloglucan endotransglucosylase/hydrolase 22*, *65-kDa microtubule-associated protein*, *Xyloglucan endotransglucosylase/hydrolase 2*, *N-glycosylase/DNA lyase OGG1* and *Cellulose synthase A catalytic subunit 2* transcripts have a greater association with vigor in soybean seeds (Figure 7 and Figure 8). To explain these results, it is necessary to consider that the germination process has several initial events and checkpoints that need to be completed for germination to occur normally [22]. In this context, a vigorous seed completes these stages more quickly, completing the program sooner than a less vigorous seed [7] as showed by our t50 data (Figure 2B,C). Based on that, the increase in the expression of *Expansin-like A1*, *Xyloglucan endotransglucosylase/hydrolase 22*, *65-kDa microtubule-associated protein*, *Xyloglucan endotransglucosylase/hydrolase 2*, *N-glycosylase/DNA lyase OGG1* and *Cellulose synthase A catalytic subunit 2* transcripts allowed the seeds to complete the necessary steps for germination in a more efficient way, which mainly contributes to an increase in the germination speed and tolerance to artificial aging, important characteristics of seed vigor, which had contrasts in our biological material (Figure 1E and Figure 2). Thus, as the expression of the genes mentioned earlier was enhanced in more vigorous seeds, the association of these genes with vigor was evident (Figure 8).

To explain the reason for the correlation of these genes with seed vigor (Figure 7 and Figure 8), it is necessary to consider the role played by these genes during the germination process. In this sense, among the steps for efficient germination mentioned earlier, DNA repair is one the first process activated and represents an important limitation for seed vigor [23], so that the time spent for metabolic repair directly influences the seed germination rate [24]. These reports, in parallel with our results, highlights the idea that increased expression of the *N-glycosylase/DNA lyase OGG1* transcript in soybean seeds with high vigor allows the embryo to acquire germination competence earlier in this species since metabolism does not deal with the effects of DNA damage. This is supported by the rapid germination, even under adverse conditions, which occurs in vigorous seeds (Figure 2), which exhibited an increase in *N-glycosylase/DNA lyase OGG1* expression during germination. These findings explain the association of this gene expression during germination with the vigor of soybean seeds as shown by our analyses (Figure 8). Here we must emphasize that this association goes beyond rapid germination, including the ability to establish seedlings from aged seeds (Figure 1E), another face of the seed vigor. This may be linked to the role of the *N-glycosylase/DNA lyase OGG1* gene in combating DNA damage caused by reactive oxygen species as demonstrated by Chen et al., [14]. These molecules have devastating effects on seed metabolism since they are associated with lipid peroxidation, protein oxidation and damage to nucleic acids according to Li et al. [25]. These molecules are abundantly present in aged seeds. Here, we emphasize that the lipid peroxidation was high in seeds with a lower vigor level (Figure 3B). In this sense, if a possible role of this gene in stress tolerance exists, it deserves to be further studied.

In relation to the expression of genes that result in growth, here associated with seed vigor (Figure 7 and Figure 8), it is necessary to consider that the activity of proteins involved in cell wall relaxation, such as expansins, XTHs, PMEs, endo-β-mannanases and other hydrolases, results in the sliding and moving away of the cellulose microfibrils. Taken together, the synchronous actions of these processes allow cell elongation, which determines radicle protrusion [18]. In soybean embryos, a radicle-derived growth pattern occurs, although the hypocotyl represents most of the cell elongation [19] and our embryonic length measurement showed that the growth occurred in a similar way (see the growth behavior of the embryonic axis on Figure 4), regardless of the different vigor of our samples (Figure 1E, Figure 2 and Figure 3). However, there is an undeniable association between *Expansin-like A1*, *Xyloglucan endotransglucosylase/hydrolase 22*, *65-kDa microtubule-associated protein*, *Xyloglucan endotransglucosylase/hydrolase 2* and *Cellulose synthase A catalytic subunit 2* expression with the embryonic axis growth, jointly with other vigor parameters here evaluated (Figure 7 and Figure 8). The explanation for this is that the advanced molecular stage through the increase in the level of transcription of the genes mentioned earlier was decisive for the manifestation of vigor in soybean seeds even if not noticeable in the embryonic axis growth in the evaluated points. Even so, it can be inferred that an increase in the *Expansin-like A1*, *Xyloglucan endotransglucosylase/hydrolase 22*, *65-kDa microtubule-associated protein*, *Xyloglucan endotransglucosylase/hydrolase 2* and *Cellulose synthase A catalytic subunit 2* expression ensures the fast germination under suboptimal conditions (Figure 2) and the ability to form normal seedlings after exposure to accelerated deterioration imposed by accelerated ageing test in high vigor seeds (Figure 1E) as a reflection of their contribution on vigor. [26] verified in *Arabidopsis* seeds that the overexpression of the *AtEXP2* gene leads to higher germination speed. It was demonstrated that submodules related to genes of the expansin group have a greater contribution in the hypocotyl elongation in soybean seeds [27], an important step in the construction of young plants in post germination events. These results permeate our findings and support us accordingly.

Our results demonstrated the assertiveness of our hypothesis of associating genes related to the growth of the embryonic axis with the manifestation of vigor, here evaluated by germination speed, stress tolerance, organ elongation of seedlings, membrane integrity and tissue deterioration. These associations have a significant contribution since seed scientists have sought to associate functional characteristics of germination to the events following this. In previous studies, we demonstrated that using biophysics signals it is possible to predict superior seedling formation [28] and that the process that occurred during germination *sensu stricto* has a positive influence in greater seedling establishment [6]. However, up to now, the contribution of gene expression on vigor has not been explored in soybean seeds, which highlights the contribution of this work to seed science and technology.

Thus, here we increase previous knowledge by demonstrating that *Expansin-like A1*, *Xyloglucan endotransglucosylase/hydrolase 22*, *65-kDa microtubule-associated protein*, *Xyloglucan endotransglucosylase/hydrolase 2*, *N-glycosylase/DNA lyase OGG1* and *Cellulose synthase A catalytic subunit 2* expression during germination contributes significantly to vigor in soybean (our study measured not only seedling performance but also germination speed, artificial aging, and biochemical properties). We noticed that this link occurred through an increase in the level of these transcripts in seeds with high vigor (Figure 5). We would like to mention that other genes related to the germination process can be analyzed to increase the range of targets in future research. Taken together, our results allow us to suggest that the expression of the genes mentioned earlier can be used to study vigor at the molecular level.

## 4. Conclusions

The gene expression of the *Expansin-like A1*, *Xyloglucan endotransglucosylase/hydrolase 22*, *65-kDa microtubule-associated protein*, *Xyloglucan endotransglucosylase/hydrolase 2*, *N-glycosylase/DNA lyase OGG1* and *Cellulose synthase A catalytic subunit 2* is associated with seed vigor in soybean seeds. Besides the use to study the vigor at the molecular level, our findings open the possibility of using the expression of the genes reported here to analyze seed vigor in soybean commercial seed lots, which should be validated for a greater number of genotypes and seed lots in future research. This use can be a strong tool for the diagnosis of vigor by the seed industry.

## 5. Materials and Methods

### 5.1. Seed Samples

Seeds of two commercial cultivars (BRS133-cv1 and MG/BR 46 Conquista-cv2) were used in this study. These cultivars have different tolerance to the production environment and are constantly propagated by our seed laboratory team. The distinct tolerance mentioned earlier allows obtaining different levels of natural vigor throughout the seasons and years. In each production season the seeds were collected manually at the mature stage (R9) according to criteria described in Basso et al. [11] and the immature, green and unformed seeds were removed from the seed lots. We screened and separated two lots (lot 1 and 2) of each cultivar produced in separate areas in Botucatu, São Paulo State, Brazil, during the 2014/15 crop season (data on environmental conditions of each area are not presented here). The screening was based on a natural difference in their seed vigor according to the results of different vigor tests. The divergence in the seed vigor in these samples would allow us to test our hypothesis. The seeds were stored at 12 °C and 50% relative humidity (RH) until the beginning of the experiments, which was not longer than fifteen days.

### 5.2. Physiological Assays

Seeds were evaluated for their physiological and biochemical properties as described below.

Water content: determined using two replicates of 20 seeds each by oven method at 105 °C for 24 h. The calculation was done on a wet basis, with the level of moisture expressed as a percentage according to the Rules for Seed Testing [29].

Normal seedlings: four replicates of 25 seeds were germinated on paper rolls moistened with water at 2.5 times the paper dry weight at 25 °C in the dark. The count of normal seedlings (here considered those with aerial part and main root ≥5 cm) was carried out after eight days according to the Rules for Seed Testing [29] criteria. The results were expressed in percentage of normal seedlings.

Time to 50% of root protrusion (t50): four replicates of 25 seeds were germinated on a paper roll as described earlier, but with the following variations. One was germinated at 25 °C, one at 15 °C, one at 10 °C, and one at 25 °C using a saline solution of NaCl at a concentration of 100 mmol.L^−1^. Every six hours, the seeds that presented root protrusion ≥ 2 mm were computed as germinated, and the t50 was calculated using the curve fitting module of the Germinator software [30].

Embryonic axis length: three replicates of 10 seeds were germinated as described by the normal seedling-test, and their embryonic axis were collected at six and 10 h. In parallel, the embryonic axis of the dry seeds were collected. The embryonic axis were photographed under a blue background and their length was measured using ImageJ software [31].

Length and dry mass of seedlings: four replicates of 10 seeds were placed longitudinally in the upper third of paper roll, which was moistened 2.5 times the paper dry weight and kept at 25 °C [32]. Eight days after germination, the seedlings were measured (cm), placed at 60 °C for 72 h hours and the dry mass results expressed in milligrams [33].

Seedling emergence: four replicates of 50 seeds were sown in sand at a depth of 3 cm and irrigated whenever necessary. The seedling count was carried out fifteen days after sowing and the results expressed in percentage [32].

Accelerated aging with NaCl saline solution (SSAA): seeds were placed in a single layer on a metallic screen suspended inside plastic boxes (11 cm × 11 cm × 3 cm) over a saturated saline solution of NaCl (75% RH) and kept at 41 °C for 72 h. After this period, the seeds were germinated as described by the normal seedling-test, and the percentage of normal seedlings was counted on the fifth day after the installation of the test [34].

Electrical conductivity: this test was performed with four replicates of 50 seeds. Seeds were placed in plastic containers with 75 mL of distilled water and kept at 25 °C for 24 h. After this period, the electrical conductivity of the solutions was performed using a conductivity meter (Digimed D31) and the results expressed in μS.cm^−1^. g^−1^ [35].

Malondialdehyde (MDA): this estimate was determined from four 100 mg seed samples macerated and homogenized in 1 mL of 50 mM phosphate buffer (pH 7.0) containing 0.67% TCA, followed by centrifugation at 15,000 *g* for 15 min. To 1.0 mL of the supernatant, 2.0 mL of 0.5% thiobarbituric acid (TBA) was added in 20% TCA. The mixture was heated at 95 °C for 30 min in a water bath and then cooled in ice. Afterwards, centrifugation was carried out at 15,000 *g* for 20 min, followed by the absorbance of the supernatant at 532 nm. The value for the nonspecific absorption of each sample at 600 nm was also recorded and subtracted from the absorbance recorded at 532 nm.

### 5.3. Gene Expression during Germination Sensu Stricto

Three biological replicates of 50 embryonic axis were isolated from dry seeds and from seeds germinated at six and 10 h, for each cultivar and lot. For total RNA extraction, the kit-NucleoSpin RNA Plant^®^ (Macherey-Nagel, Düren, Germany was used. For cDNA synthesis, the High-Capacity cDNA Reverse Transcription kit (Applied Biosystems, Victoria, Australia) was used. The recommendations of each manufacturer were followed to perform these steps.

The target genes were identified by literature searches for genes associated with germination, such as DNA repair, oxidative stress defense, cell respiration, reserve mobilization, cell stretching and expansion. The list of genes, their functions and the sequence of primers used are listed in Supplementary Table S1.

The genes were amplified in real time-qRT-PCR-using the KiCqStart^®^ SYBR^®^ Green qPCR ReadyMix kit (Sigma-Aldrich, St. Louis, MO, USA), in an Eco Real-Time optical thermal cycler (Illumina, San Diego, CA, USA), and the data was analyzed using Illumina’s EcoStudy v5.0 software. Relative quantification (RQ) was determined by the 2ˆ^−∆∆Ct^ method [36], using two reference genes, Importin beta-2 subunit family protein (Glyma.20G106300) and 20S proteasome subunit beta (Glyma.06G078500) [37].

### 5.4. Statistical Design

The physiological properties were subjected to analysis of variance, and when significant, the means were compared by the Tukey test at 0.05 confidence level. The relative gene expression data were obtained using the REST^®^ program, which performs the comparative quantification by the method of “Pair-Wise Fixed Reallocation Randomization Test” [38]. The dry seeds from lot 1 of each cultivar were adopted as a control group to perform this calculation.

The subsequently analyses were performed using the R programming language v4.1.0 through the RStudio integrated development environment platform v1.4.1717. For the analysis of correlations between gene expression and physiological assays, two techniques were used jointly. The non-parametric bootstrap resampling method, which computationally generated 10,000 new combinations of data from random iterative selections from the original dataset. Then, for each resampling, the correlation between the 2 groups of variables (gene expression and physiological properties) was measured. This measurement was found by means of the canonical regulatory correlation (RCCA) analysis of the CCA::rcc (...) library and function. As described by Leurgans et al. [39] and Vinod [40], this is a process similar to ridge regression, as the RCCA uses an estimated constant to penalize the covariance matrices that are used for the calculation, avoiding problems of high linear dependence. Therefore, 10,000 measurements of the first (most expressive) eigenvalue of the RCCA were obtained and characterized by means of a Boxplot graph. For the analysis of clusters of variables, the ClustOfVar library was used with the hclustvar (...) function. This is due to the objective of finding similarity between variables, different from the usual method of clustering between observations that uses distances (e.g., Euclidean). The similarity measurement used in the work is based on the first principal components of the decomposition of singular values of the PCAmix method (Main Components Method for mixing quantitative and qualitative variables), and thus through a homogeneity criterion measured by the square of the measure of Pearson’s correlation, it was possible to characterize the greatest relationship between groups of variables [20].

## Data Availability

The datasets analyzed during the current study are available from the corresponding author upon reasonable request.

## Supplementary Materials

The following are available online at https://www.mdpi.com/article/10.3390/plants11101310/s1. Table S1: Genes (mRNAs) studied in the embryonic axis of soybean seeds during germination *sensu stricto* and primers used for real-time PCR reactions. Figure S1: First eigenvalue of the regulatory canonical correlation (RCCA) from the Bootstrap 10,000 of gene expression at 6 h and 10 h during germination *sensu stricto* and vigor properties.
