# Supplementary material for: Transcripts Expressed during Germination Sensu Stricto Are Associated with Vigor in Soybean Seeds
Source: Plants (Basel). 2022 May 14;11(10):1310. doi: 10.3390/plants11101310 (PMC9147077; doi:10.3390/plants11101310)
Supplement: Supplementary file 1 [file plants-11-01310-s001.zip › Figure S1.pdf]

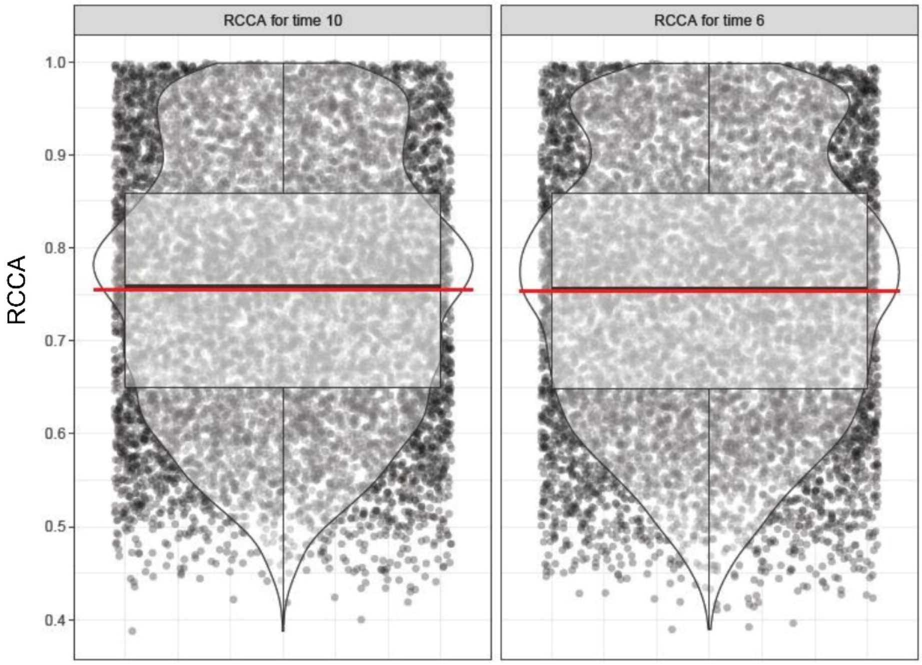

Supplementary figure S1. First eigenvalue of the regulatory canonical correlation (RCCA) from the Bootstrap 10,000 of gene expression at 6 and 10h during germination *sensu stricto* and vigor properties. The red line indicates de average value of the correlation.
